# Anti-inflammatory Effects of Perioperative Dexmedetomidine Administered as an Adjunct to General Anesthesia: A Meta-analysis

**DOI:** 10.1038/srep12342

**Published:** 2015-07-21

**Authors:** Bo Li, Yalan Li, Shushi Tian, Huixia Wang, Hui Wu, Aihua Zhang, Chengjie Gao

**Affiliations:** 1 Department of Anesthesiology, Jinan General Hospital, PLA Jinan Military Area Command, Jinan 250031, Shandong, China; 2 Department of Anesthesiology, The First Affiliated Hospital of Jinan University, Guangzhou 510632, Guangdong, China; 3 Department of Radiology, No. 261 Hospital of PLA, Beijing, China; 4 Department of Anesthesiology, The People’s Hospital of Zhangqiu, Zhangqiu 250200, Henan, China; 5 Department of Anesthesiology, Yancheng City No.1 People’s Hospital, Yancheng 224005, Jiangsu, China

## Abstract

The aim of this meta-analysis is to examine the effects of dexmedetomidine on serum inflammatory markers when administered perioperatively. We searched multiple electronic databases for relevant research papers, and carried out meta-analyses of weighted mean differences and interpreted in the light of statistical heterogeneity (I^2^). Fifteen RCTs recruiting 641 patients were included. Dexmedetomidine treatment significantly decreased interleukin-6 (IL-6), IL-8 and tumor necrosis factor-alpha (TNF-α) levels with mean differences [95% CI] in the changes from baseline between dexmedetomidine treated and controls of −25.14 [−35.29, −15.00]; P < 0.00001 (for IL-6), −5.69 [−10.77, −0.60]; P < 0.04 (for IL-8), and −20.30 [−30.93, −9.67]; P < 0.0002 (for TNF-α) immediately after surgery; and −41.55 [−57.41, −25.70]; P < 0.00001 (IL-6), −6.46 [−10.83, −2.08]; P < 0.005 (IL-8), and −14.67 [−22.61, −6.73]; P < 0.0003 (TNF-α) on postoperative day 1 (random effects). IL-10 levels were found to increase significantly a day after surgery (8.33 [3.31, 13.36]; P = 0.001). Subgroup analyses did not reveal significant differences. In conclusion, perioperative adjunctive use of dexmedetomidine substantially decreases serum IL-6, IL-8 and TNF-α levels.

The postoperative period constitutes a regenerative period associated with remarkable physiological processes for wound healing. Among these, inflammation is an important process which sets a stage for the protection against infections and regenerative cell recruitment^1^. However, excessive inflammatory response leaves deleterious effects not only on wound healing but also on other physiological functions, therefore, a balance between inflammatory factors and timely resolution of inflammation is essential for wound healing and normalization^2^. Anesthetics favoring this delicate balance are thus desirable as their use may reduce postoperative complications and mortality. Among such anesthetics, dexmedetomidine has emerged as an important modulator of anxiolysis that tends to normalize arterial blood pressure and heart rate without declining respiratory function or arterial oxygen saturation^3^,^4^,^5^. There is considerable evidence that this α2-adrenergic receptor agonist can manifest additional protective effects on the heart, brain and kidneys^6^,^7^,^8^,^9^.

Preclinical studies on murine models have revealed that dexmedetomidine manifests multi-faceted protective effects including the inhibition of proinflammatory cytokine production^10^,^11^,^12^. *In vitro* studies with human whole blood samples have shown that dexmedetomidine significantly suppresses lipopolysaccharide induced proinflammatory mediators including tumor necrosis factor - alpha (TNF-α), interleukin (IL)-6, and IL-8 production dose-dependently^13^. Clinical investigations with human subjects evaluating the effects of dexmedetomidine on serum inflammatory cytokines during perioperative conditions are rather fragmentary with between-study inconsistencies in the outcomes^14^,^15^,^16^,^17^,^18^,^19^,^20^,^21^,^22^,^23^,^24^,^25^,^26^,^27^,^28^. In order to evaluate the effect of dexmedetomidine on inflammatory responses, we performed a meta-analysis of randomized controlled trials (RCTs) that examined one or more parameters of inflammatory markers (pro-inflammatory as well as anti-inflammatory) in patients treated with dexmedetomidine as an adjunct to general anesthesia during surgical/critical procedures.

## Method

### Literature search

The literature was searched for relevant peer-reviewed research papers published between 1990 and July 2014. Electronic databases including EBSCO, Embase, Google Scholar, Ovid SP, PubMed, Scopus, and Web of Science were utilized. The major MeSH terms and important keywords including dexmedetomidine, anesthesia, sedation, surgery, inflammation, anti-inflammatory, pro-inflammatory cytokine, interleukin, IL-1, IL-2, IL-4, IL-6, IL-8, IL-10, IL-12, IL-18, tumor necrotic factor-α (TNF-α), and c-reactive protein (CRP) were used. Reference lists of significant articles were scrolled and corroborations were also explored.

### Inclusion and exclusion criteria

The inclusion criteria were: (a) trials of patients undergoing surgical procedures or traumatic interventions wherein the effectiveness of perioperative dexmedetomidine against inflammatory stress was compared to a control group; (b) trials investigated the effect of dexmedetomidine on inflammatory response by measuring one or more relevant pro-inflammatory and/or anti-inflammatory cytokines, and other important modulators of inflammation; and (c) trials reporting at least one outcome measure of inflammatory response after examining baseline, postoperative, and late-point samples. Exclusion criteria were: (a) trials assessing the effects of dexmedetomidine on hemodynamic indicators or injury markers other than inflammatory modulators; (b) single arm studies; (c) case reports; and (d) *in vitro* studies.

### Primary and secondary data points

The primary endpoints of the present study were changes in the serum proinflammatory cytokines, IL-2, IL-6, IL-8, IL-12, IL-18, TNF-α, and anti-inflammatory cytokines (IL-1, IL-4, IL-10, and IL-16) levels from baseline. The secondary endpoint was the changes in serum CRP levels.

### Quality assessment of the trials

Each individual study was carefully evaluated for strengths, limitations, design, methodology, outcome dissemination, and interpretation. A formal quality assessment was made by using the Cochrane Collaboration Risk of Bias Assessment Tool for the assessment of RCTs which examines each trial’s internal validity, possibilities of risk of bias in various phases of trial conduct and outcome analyses. The tool weighs risk of a possible bias under “Low” or “High” measures and does not attempt numerical scores^29^.

### Data collection and statistical analysis

The required data including primary and secondary endpoint outcomes, dexmedetomidine dosage, general anesthetic type and dosage, subjects’ demographics, and other relevant information was collected from the individual papers and organized in a database by two authors (BL and HW) independently with good inter-rater reliability (kappa = 0.95).

Meta-analyses were performed using the RevMan software (Version 5.2; Cochrane Collaboration) under both fixed- (FEM) and random-effects (REM) models. Changes from baseline in serum inflammatory markers were first measured and then means and standard deviations were used to calculate mean differences with 95% confidence intervals (CI). For studies reporting effect sizes as a median with range or interquartile range, the median was used instead of the mean while the standard deviation was estimated from range or interquartile range with the help of statistical tools providing reliable approximations^30^,^31^. The overall effect of each meta-analysis was a weighted average with 95% CI of the inverse variance adjusted effect size of individual studies.

Between-studies statistical heterogeneity was tested with I^2^ index. Sensitivity analyses were performed to examine the source of higher heterogeneity and effect size validity. Subgroup analyses were carried out in order to evaluate the impact of dose concentration, duration of surgery, duration of anesthesia, timing of dexmedetomidine administration, and type of surgery on the changes from baseline in the serum levels of IL-6 and TNF-α after surgery. For each variable, subgroup pair was first defined and then meta-analyzed. The overall effect sizes of each member of a subgroup pair were subjected to two-tailed z test for examining the significance of difference. Details of these subgroups are given in Table 1.

## Results

Fifteen RCTs^14^,^15^,^16^,^17^,^18^,^19^,^20^,^21^,^22^,^23^,^24^,^25^,^26^,^27^,^28^ were selected for inclusion. The process of study screening and selection is summarized in Fig. S1. Multiple database literature searches led to the identification of 1149 article abstracts. At the screening level, 818 search engine-retrieved abstracts were excluded because of the irrelevancy with research question. Main reasons for the exclusions at study eligibility determination stage included: the endpoints restricted to sympatho-adrenal parameters only (n = 21); the endpoints restricted to hemodynamic, cardiovascular, respiratory, neurological, and/or other manifestations of physiological stress (n = 87); the study performed by using animal model/s (n = 46); other dexmedetomidine efficacy, safety, comparative, dose-sparing assessments and combinational studies (n = 162).

Major characteristics of the included studies are presented in Table S1. From the included studies, data of 641 patients with average age 47.47 ± 8.47 years (range: 4 ± 5 months to 72.7 ± 7 years) were used for the meta-analyses of various parameters. Initial doses (first 10 minutes) of dexmedetomidine ranged from 0.5 to 2.5 μg/kg (mean: 1.08 ± 0.56) which followed maintenance doses of 0.05–0.5 μg/kg/h (mean ± SD: 0.36 ± 0.15) throughout anesthesia period. Dexmedetomidine was administered intraoperatively in 12 studies, postoperatively in two studies, and for sedation of trauma patients in one study. Anesthetics and anxiolytics administered during the surgical procedures included sufentanil, fentanyl, alfentanil, diazepam, propofol, midazolam, lidocaine, etomidate, bupivacaine, sevoflurane, isoflurane, vecuronium, pancuronium, rocuronium, vecuronium, and atracurium (Table S1).

Types of surgeries included elective multilevel lumbar fusion, hepatectomy, laparoscopic cholecystectomy, tetralogy of fallot, ventricular septal/atrioventricular defects, elective major abdominal/pelvic surgery, elective multilevel lumbar fusion, one lung ventilation, cardiopulmonary bypass, ileus, esophageal carcinoma resection, and cerebral surgery.

The quality of the included studies, in general, ranged from medium to high, when weighed with the Cochrane Collaboration Risk of Bias Assessment Tool (Table S2). Among the included studies, all were randomized but 7 were double blind, one single blind, and remainder not blinded interventions.

**Figure 1:**
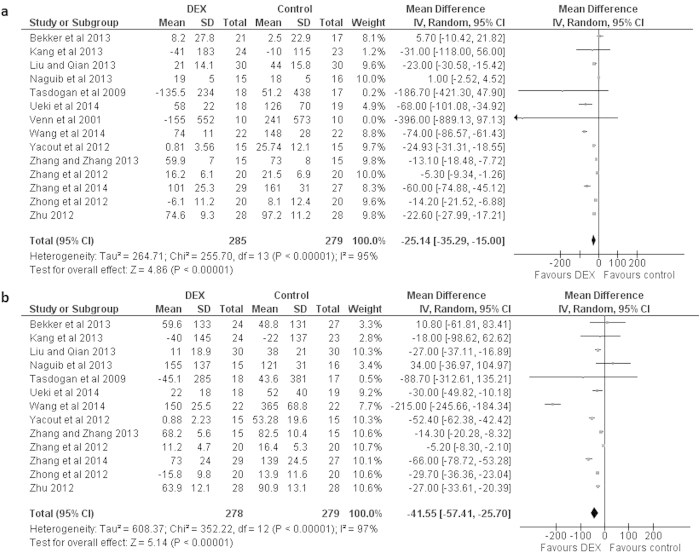
Forest graph showing the effect of dexmedetomidine on the changes from baseline in serum IL-6 levels (**a**) after surgery, and (**b**) a day after surgery. Boxes represent mean differences and line across each box represents respective 95% CI. Diamond represents overall effect size of the meta-analysis.

Major findings of the meta-analyses are presented in Table 1. Dexmedetomidine administration significantly decreased IL-6 levels in the samples taken from patients after surgery. Mean difference [95% CI] between dexmedetomidine treated and control patients in changes from baseline was −25.14 [−35.29, −15.00] pg/ml; *P* < 0.00001; REM (Fig. 1a). This statistically significant difference was also observed on the first postoperative day with a mean of −41.55 [−57.41, −25.70] pg/ml; *P* < 0.00001; REM (Fig. 1b).

In the subgroup analyses, there were no significant differences between the utility of dexmedetomidine dose of over median vs under median; or surgery duration of more than median vs less than median; or anesthesia duration of more than median vs less than median; or timing of dexmedetomidine administration (intraoperative vs postoperative); or surgery type of cardiac vs non-cardiac/gastrointestinal vs non-gastrointestinal (Table 2).

**Figure 2:**
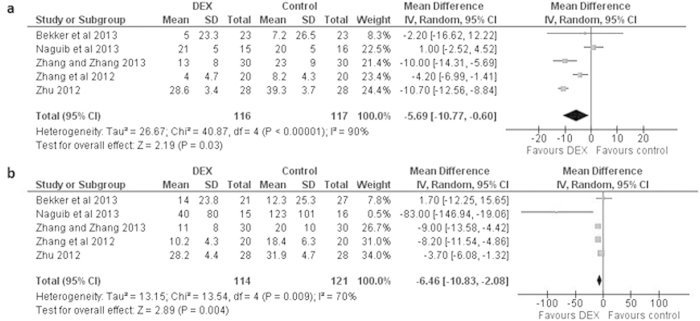
Forest graph showing the effect of dexmedetomidine on the changes from baseline in serum IL-8 levels (**a**) after surgery, and (**b**) a day after surgery. Boxes represent mean differences and line across each box represents respective 95% CI. Diamond represents overall effect size of the meta-analysis.

Among the other proinflammatory markers, IL-8 levels also decreased significantly in the postoperative period as well as a day after surgery in dexmedetomidine administered patients with a mean difference of −5.69 [−10.77, −0.60] pg/ml (*P* < 0.04) immediately postoperative and −6.46 [−10.83, −2.08] pg/ml (*P* < 0.005) at postoperative day 1 (Fig. 2a,b).

**Figure 3:**
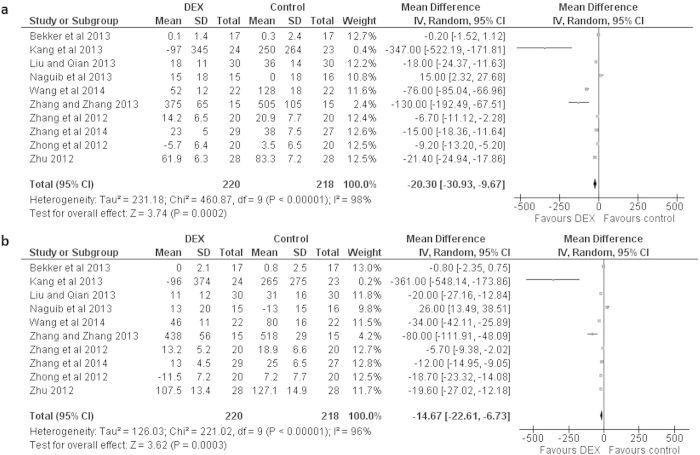
Forest graph showing the effect of dexmedetomidine on the changes from baseline in serum TNF-α levels (**a**) after surgery, and (**b**) a day after surgery. Boxes represent mean differences and line across each box represents respective 95% CI. Diamond represents overall effect size of the meta-analysis.

Dexmedetomidine administration was also associated with a significant reductions in the serum concentrations of TNF-α by a mean difference (95% CI) between dexmedetomidine treated and control patients in changes from baseline of −20.30 [−30.93, −9.67] pg/ml (*P* < 0.0002; REM) immediately after surgery and −14.67 [−22.61, −6.73] pg/ml (*P* < 0.0003; REM) one day after surgery (Fig. 3a,b).In the subgroup analyses, there were no significant differences between the utility of dexmedetomidine dose of over median vs under median; or surgery duration of more than median vs less than median; or anesthesia duration of more than median vs less than median; or surgery type of cardiac vs non-cardiac/gastrointestinal vs non-gastrointestinal (Table 2).

There was no significant difference in the changes from baseline in the serum levels of IL-10 between dexmedetomidine treated and control patients soon after surgery (mean difference −2.18 [−31.28, 26.92]; P = 0.88; REM) but IL-10 levels were significantly higher in dexmedetomidine treated patients a day after surgery (mean difference 8.33 [3.31, 13.36]; P = 0.001; FEM). No significant differences were noted with regards to CRP levels (Table 1).

For the evaluation of other inflammatory cytokines, data were either not available or insufficient for the meta-analyses. Among these, no significant differences were noted in the changes from baseline either immediately after surgery or a day after surgery between dexmedetomidine and control patients in the levels of IL-1a^14^, IL-1b^15^, and IL-1^19^,^25^. However, postoperative IL-4 levels were found to decrease significantly in the dexmedetomidine treated subjects compared to saline-treated controls in one study (mean difference in the change from baseline: −260.00 [−420.39, −99.61]; P < 0.002)^16^.

## Discussion

The surgical stress response involves metabolic, inflammatory, and immune reactions. Proinflammatory cytokines and chemokines attract leukocytes to the inflammatory sites and recruit neutrophils for the phagocytosis of bacteria. Increased secretion of cytokines as a result of stimulation of immunological and inflammatory reactions disturbs normal proinflammatory and anti-inflammatory cytokine balance which can increase morbidity and mortality^32^. Sedatives can reduce the severity of this surgical stress response and improve the tolerance of patients for critical procedures^33^, and of the modern sedatives, dexmedetomidine can provide multiple benefits^1^,^2^,^3^,^4^,^5^,^6^,^7^.

This meta-analysis was performed to synthesize the fragmentary data pertaining to the effects of perioperative use of dexmedetomidine on inflammatory modulators. We have found that when used as an adjunct to general anesthesia, dexmedetomidine significantly reduced postoperative serum IL-6, IL-8, and TNF-α levels postoperatively as well as at postoperative day 1, and significantly increased IL-10 levels a day after surgery.

Interleukin-6 is a proinflammatory cytokine that exerts both local and systemic effects for reducing tissue injury and combating infections while promoting biochemical mechanisms of healing and repair^34^. It is the principal interleukin released after surgery and acts as a reliable indicator of the inflammatory response to surgical trauma^35^. Tumor necrosis factor-α is also a proinflammatory cytokine that regulates several other cytokines^36^. Increased production of TNF-α is associated with several chronic inflammatory conditions^37^. Interleukin-8, another pro-inflammatory cytokine, has also been implicated in several pathological conditions including some cancers and may act as a prognostic or predictive biomarker^38^,^39^. In the present study, we find a significant decrease in IL-6, IL-8 and TNF-α levels in dexmedetomidine treated patients which is indicative of the anti-inflammatory potentials of dexmedetomidine when used as perioperative adjunctive drug.

Interleukin-10 is an anti-inflammatory cytokine. In the present study, whereas, IL-10 levels were non-significantly higher in the dexmedetomidine treated patients soon after surgery, but a day after surgery, there was a significant increase in IL-10 levels in dexmedetomidine treated than in control patients. However, this meta-analytical outcome gained almost all weight from 2 studies^14^,^25^ and therefore, this outcome is subject to the availability of more data in future.

Interleukin-6, IL-8 and TNF-α are implicated in the exhibition of sickness behavior^40^,^41^. Dexmedetomidine is reported to attenuate sepsis-induced lung and kidney injuries in a rodent model^42^. Keeping in view these observations and in the light of results of several studies including meta-analyses that report significantly shorter ICU stay of dexmedetomidine treated patients in perioperative conditions^43^,^44^,^45^, it seems reasonable to ascribe the anti-inflammatory effects of perioperative dexmedetomidine observed in this meta-analysis as beneficial. Indeed, a pre-emptive administration of dexmedetomidine has been advocated by some authors keeping in view its anti-inflammatory effects^15^,^46^.

Effects of anesthetics on immunomodulation of inflammation follow a delicate pattern as the immunosuppression can leave positive as well as negative effects and therefore it is necessary to apply an anesthetic or anesthetics in the light of the immunological status of patients. Hazards associated with perioperative immunosuppression can be the increased risk of tumor metastasis and infection whereas the anti-inflammatory effects of anesthetics may provide more benefit in conditions such as ischemia, reperfusion injury and systemic inflammatory response syndrome^47^.

Although a number of mechanisms of dexmedetomidine action have been postulated including the modulation of cytokine production by macrophages and monocytes during the stress response which may also be stimulated via the α2-adrenoceptors, inhibition of apoptosis, central sympatholytic effects including the stimulation of cholinergic anti-inflammatory pathway, and antinociceptive action involving interactions between pain and immune factors such as proinflammatory cytokines^15^, however, so far, it is not clear what mechanisms are involved in the perioperative effectiveness of dexmedetomidine rather further research is needed.

The main limitation of this study was the limited number of papers that could be included in some meta-analytical comparisons, and therefore, evidence regarding the effects of dexmedetomidine on IL-1, IL-2, IL-4, and CRP levels remains inconclusive. Methodological heterogeneity may also have some impact on overall outcomes; eleven of the included studies administered dexmedetomidine intraoperatively, 2 studies administered postoperatively, and one study administered the drug to severe trauma patients. One study recruited pediatric patients which was included in the meta-analysis keeping in view that the patterns of cytokine secretion in children remain similar to adults^48^,^49^. Use of different anesthetics in different studies may also have slight interference in manifesting the effects of dexmedetomidine. However, sensitivity analyses did not exhibit any significant difference in the overall results.

## Conclusion

Perioperative use of dexmedetomidine as an adjunct to general anesthesia leads to significant decreases in serum levels of IL-6, IL-8, and TNF-α within a period of 24 hours postoperatively. High level of meta-analytical heterogeneity may have some impact on the overall results achieved herein. More studies will be required to further refine these results and to evaluate the effect of perioperative dexmedetomidine on IL-1, IL-2, IL-4, IL-10, IL-18, and CRP.

## Additional Information

**How to cite this article**: Li, B. *et al.* Anti-inflammatory Effects of Perioperative Dexmedetomidine Administered as an Adjunct to General Anesthesia: A Meta-analysis. *Sci. Rep.* **5**, 12342; doi: 10.1038/srep12342 (2015).

## Supplementary Material

Supplementary Information

## Figures and Tables

**Table 1:** Meta-analyses outcomes as mean differences and 95% confidence interval between dexmedetomidine treated and control patients in changes from baseline in inflammatory markers.

Parameter	Studies	Patients	Mean difference [95% confidence interval]		I^2^	Results favour
			**Fixed effects**	**Random effects**		
IL-6 levels (pg/ml) after surgery	14	564	−12.32 [−14.19, −10.46]; P < 0.00001	−25.14 [−35.29, −15.00]; P < 0.00001	95%	DEX
IL-6 levels (pg/ml) a day after surgery	13	557	−18.39 [−20.54, −16.25]; P < 0.00001	−41.55 [−57.41, −25.70]; P < 0.00001	98%	DEX
IL-8 levels (pg/ml) after surgery	5	233	−7.36 [−8.70, −6.02]; P < 0.00001	−5.69 [−10.77, −0.60]; P < 0.04	90%	DEX
IL-8 levels (pg/ml) a day after surgery	5	235	−5.73 [−7.50, −3.96]; P < 0.00001	−6.46 [−10.83, −2.08]; P < 0.005	70%	DEX
TNF-α levels (pg/ml) after surgery	10	438	−6.00 [−7.05, −4.94]; P < 0.00001	−20.30 [−30.93, −9.67]; P < 0.0002	98%	DEX
TNF-α levels (pg/ml) a day after surgery	10	438	−5.90 [−7.08, −4.71]; P < 0.00001	−14.67 [−22.61, −6.73]; P < 0.0003	96%	DEX
IL-10 levels (pg/ml) after surgery	4	153	4.44 [−0.80, 9.68]; P = 0.1	−2.18 [−31.28, 26.92]; P = 0.88	89%	Indifferent
IL-10 levels (pg/ml) a day after surgery	3	158	8.33 [3.31, 13.36]; P = 0.001	8.08 [−0.06, 16.22]; P = 0.05	45%	DEX
CRP levels (mg/l) after surgery	2	94	1.07 [−0.74, 2.88]; P = 0.25	−23.05 [−73.86, 27.75]; P = 0.37	94%	Indifferent
CRP levels (mg/l) a day after surgery	2	94	8.34 [2.31, 14.37]; P < 0.008	−49.58 [−180.90, 81.73]; P = 0.46	99%	Indifferent

**Table 2:** Subgroup analyses for the assessment of impact of dexmedetomidine (DEX) dose, operative time, duration of anesthesia, timing of DEX administration and surgery type on serum IL-6 and TNF-α levels after surgery.

**Subgroups**	**Studies with**	**No. of studies**	**Mean difference [95% CI] between DEX treated and control subjects**	**Between subgroup significance**
**IL-6**				
D1	DEX dose* equal & over median (0.4 μg/kg/h)	8	−17.54 [−28.57, −6.52]; P = 0.002; REM	Z = 0.656; P = 0.512
D2	DEX dose* under median (0.4 μg/kg/h)	6	−91.66 [−126.28, −57.04]; P < 0.00001; REM	
ST1	Surgery duration over median (184 min)	5	−31.31 [−54.82, −7.81]; P = 0.009; REM	Z = 0.041; P = 0.967
ST2	Surgery duration under median (184 min)	4	−30.01 [−46.69, −13.33]; P = 0.0004; REM	
AT1	Anesthesia duration over median (266 min)	5	−108.29 [−171.25, −45.32]; P = 0.0008; REM	Z = 0.919; P = 0.358
AT2	Anesthesia duration under median (266 min)	4	−39.16 [−60.86, −17.46]; P = 0.0004; REM	
IO	Intraoperative DEX administration	11	−25.86 [−36.70, −15.02]; P < 0.00001; REM	Z = 0.635; P = 0.525
PO	Postoperative DEX administration	3	−199.00 [−506.41, 108.41]; P = 0.20; REM	
C	Cardiac surgery patients	4	−18.50 [−33.48, −3.52]; P = 0.02; REM	Z = 1; P = 0.318
NC	Non-cardiac surgery patients	10	−54.08 [−74.05, −34.11]; P < 0.00001; REM	
GIT	Gastrointestinal surgery patients	5	−113.58 [−167.78, −59.38]; P < 0.0001; REM	Z = 1.75; P = 0.079
NGIT	Non-gastrointestinal surgery patients	9	−13.64 [−21.95, −5.33]; P = 0.001; REM	
**TNF-α**				
D1	DEX dose* equal & over median (0.4 μg/kg/h)	5	−8.88 [−19.96, 2.20]; P = 0.12; REM	Z = 0.983; P = 0.325
D2	DEX dose* under median (0.4 μg/kg/h)	4	−43.26 [−69.92, −16.60];p = 0.001; REM	
ST1	Surgery duration over median (167 min)	4	−26.08 [−45.08, −7.09]; P = 0.007; REM	Z = 0.923; P = 0.35
ST2	Surgery duration under median 167 min)	3	−134.65 [−265.76, −3.54]; P = 0.04; REM	
AT1	Anesthesia duration over median (226 min)	4	−23.75 [−41.44, −6.07];P = 0.008; REM	Z = 0.642; P = 0.52
AT2	Anesthesia duration under median (226 min)	2	−171.94 [−490.11, 146.24]; P = 0.29; REM	
C	Cardiac surgery patients	3	−25.76 [−62.05, 10.52]; P = 0.16; REM	Z = 0.095; P = 0.924
NC	Non-cardiac surgery patients	7	−22.32 [−34.65, −9.98]; P = 0.0004; REM	
GIT	Gastrointestinal surgery patients	3	−72.86 [−131.99, −13.74]; P = 0.02; REM	Z = 1.177; P = 0.239
NGIT	Non-gastrointestinal surgery patients	7	−9.79 [−18.79, −0.80]; P = 0.03; REM	

^*^Maintenance dose
