# Supplementary material for: Anti-inflammatory Effects of Perioperative Dexmedetomidine Administered as an Adjunct to General Anesthesia: A Meta-analysis
Source: Sci Rep. 2015 Jul 21;5:12342. doi: 10.1038/srep12342 (PMC4508837; doi:10.1038/srep12342)
Supplement: Supplementary Information [file srep12342-s1.doc]

**Title Page**

**Anti-inflammatory Effects of Perioperative Dexmedetomidine Administered as an Adjunct to General Anesthesia: A Meta-analysis**

Bo Li^1,#^, Yalan Li^2,#^, Shushi Tian^3^, Huixia Wang^1^, Hui Wu^4^, Aihua Zhang^5,*^, Chengjie Gao ^1,*^

^1^Department of Anesthesiology, Jinan General Hospital, PLA Jinan Military Area Command, Jinan 250031, Shandong, China;

^2^Department of Anesthesiology, The First Affiliated Hospital of Jinan University, Guangzhou 510632, Guangdong, China;

^3^Department of Radiology, No. 261 Hospital of PLA, Beijing, China;

^4^Department of Anesthesiology, The People's Hospital of Zhangqiu, Zhangqiu 250200, Henan, China;

^5^Department of Anesthesiology, Yancheng City No.1 People's Hospital, Yancheng 224005, Jiangsu, China

^#^Bo Li and Yalan Li contributed equally to this work.

***Corresponding authors:**

Dr. Aihua Zhang

Department of Anesthesiology, Yancheng City No.1 People's Hospital

No.16, Yuehe Road, Yancheng 224005, Jiangsu, China

Tel: +86-515-88508813, Fax: +86-515-88508813

Email: 18921885299@189.cn

Dr. Chengjie Gao

Department of Anesthesiology, Jinan General Hospital, PLA Jinan Military Area Command

No.25, Shifan Road, Tianqiao District, Jinan 250031, China

Tel: +86-531-5166-6661 Fax: +86-531-5166-6661

Email: gaochj@hotmail.com

Running title: Anti-inflammatory effects of perioperative dexmedetomidine


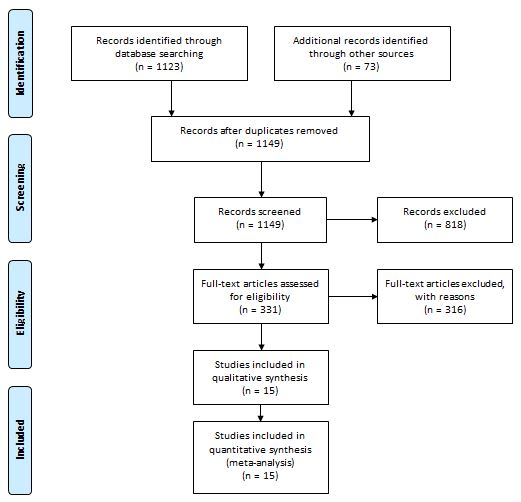


Figure S1: A flowchart of study screening and selection process.

**Table S**1: Characteristics of the included studies

| **Study/design** | **No. of patients / Age** | **Surgery / duration (DEX vs control)** | **DEX conc. / administration** | **General anesthesia / duration in minutes (DEX vs saline)** |
| --- | --- | --- | --- | --- |
| Bekker et al., 2013 [14] / DB-RCT | DEX: 26 / 55.3±11.3 yr  SAL: 28 / 57±11.1 yr | Elective multilevel lumbar fusion /  230±84.7 vs 227.3±93.4 min | 0.5 µg/ml/h / Intraoperative | Propofol (1.5–2 mg/kg), fentanyl (2–5 µg/kg) / 304±85.9 vs 295.9±102.2 min |
| Kang et al., 2013 [15] / DB-RCT | DEX: 24 / 43.4±8.6 yr  SAL: 23 / 47.7±3.9 yr | Laparoscopic cholecystectomy /  40.6±15.9 vs 38.3±19.9 min | 1 µg/kg for 10 min then 0.5 µg/kg/h / Intraoperative | Lidocaine (40 mg), propofol (2.0 mg/kg), rocuronium (1.0 mg/kg) / 57.4±15.7 vs 54.6±19.5 |
| Kim et al., 2014 [16] / SB-RCT | DEX: 23 / 44.3 ± 3.7 yr  SAL: 23 / 42.7 ± 7.7 yr | Laparoscopic cholecystectomy /  41.8±16.8 vs 44.5±13.5 min | 1 µg/kg for 10 min then 0.5 µg/kg/h / Intraoperative | Lidocaine (40 mg), propofol (2 mg/kg), rocuronium (1 mg/kg). Sevoflurane (2–5 vol% + 100% O_2_) / 55.4±16.9 vs 60.4±13.7 min |
| Liu and Qian, 2013 [17] / RCT | DEX: 30 / 57±9 yr  SAL: 30 / 55±8 yr | Cardiopulmonary bypass | 1 µg/kg for 10 min then 0.4 µg/kg/h / Intraoperative | Midazolam (0.05–0.01 mg/kg), fentanyl (5–10 µg/kg), etomidate (0.03) mg/kg and vecuronium (0.1–0.15 mg/kg) |
| Naquib et al., 2013 [18] / DB-RCT | DEX: 15 / 5 (2–21) mo  SAL: 16 / 4 (3–7) mo | Tetralogy of fallot, ventricular septal / atrioventricular defects | 1 µg/kg for 10 min then 0.5 µg/kg/h / Intraoperative | Fentanyl (10 µg/kg), Isoflurane |
| Tasdogan et al., 2009 [19] / RCT | DEX: 20 /58 (21–78) yr  SAL: 20 / 50 (19–74) yr | Post-surgery ICU patients | 1 µg/kg for 10 min then 0.2–0.25 µg/kg/h / Postoperative | Fentanyl (1–3 mg/kg), alfentanil (0.25–1 µg/kg/min) / 20±3 vs 21±3 h |
| Ueki et al., 2014 [20] / DB-RCT | DEX: 28 / 70.5±9.5 yr  SAL: 19 / 69±11.7 yr | Cardiopulmonary bypass /  192 (78–267) vs 181 (86–210) | 1 µg/kg for 10 min then 0.5 µg/kg/h / Intraoperative | Diazepam (5mg), midazolam (3–5 mg), fentanyl (30 µg/kg), sevoflurane (0.8–2%) in 40% N_2_O + O_2_ and rocuronium (0.6 mg/kg) |
| Venn et al., 2001 [21] / DB-RCT | DEX: 10; ˃ 18 yr  PROP: 10; ˃18 yr | Post abdominal/pelvic surgery patients / 330±480 vs 270±324 min | 2.5 µg/kg for 10 min then 0.2–0.25 µg/kg/h / Postoperative | Propofol (1–3 mg/kg), alfentanil (0.25–1 µg/kg/min), fentanyl / 600±567 vs 720±486 min |
| Wang et al., 2014 [22] / DB-RCT | DEX: 22 / 27–68 yr  SAL: 22 / 30–69 yr | Hepatectomy /  176±41.5 vs 193±47.1 min | 1 µg/kg for 10 min then 0.3 µg/kg/h / Intraoperative | Propofol (3–4 µg/kg), fentanyl 3 µg/kg) and cisatracurium (5–10 mg) / 241±52.3 vs 228±57.2 min |
| Yacout et al., 2012 [23] / DB-RCT | DEX: 15 / 40.6±6.5 yr  SAL: 15 / 47±6.52 yr | Elective major abdominal surgery /  194±13.3 vs 192±17.2 min | 1 µg/kg for 10 min then 0.5 µg/kg/h / Intraoperative | Standard anesthesia / 206.3±15 vs 204.67±16.95 min |
| Zhang and Zhang, 2013 [24] / RCT | DEX: 15 / 54±10 yr  SAL: 15 / 56±8 yr | Cardiopulmonary bypass /  149±34 vs 143±29 min | 1 µg/kg for 10 min then 0.05 µg/kg/h / Intraoperative | Midazolam (0.05 mg/kg), sufentanil (1 µg/kg), etomidate (0.3 mg/kg) and rocuronium (0.6 mg/kg) |
| Zhang et al., 2012 [25] / RCT | DEX: 20 / 39±15 yr  SAL: 20 / 43±13 yr | Cerebral surgery /  340±102 vs 320±113 min | 0.5 µg/kg for 10 min then 0.2 µg/kg/h /Intraoperative | Midazolam (0.05 mg/kg), fentanyl (4–6 µg/kg), propofol 1–1.5mg/kg) and vecuronium (0.1 mg/kg) / Usage: 256±102 vs 276±89 ml |
| Zhang et al., 2014 [26] / RCT | DEX: 29 / 72±7 yr  SAL: 27 / 71±6 yr | Surgery for esophageal carcinoma /  160±35 vs 171±20 min | 0.3 µg/kg for 10 min then 0.3 µg/kg/h / Intraoperative | Midazolam 0.08–0.12 mg/kg, sufentanil 0.1–1 µg/kg, etomidate 0.2–0.6 mg/kg, propofol 4–8 mg/kg/min, benzoulfonic acid and atracurium 0.15 mg/kg / 296±19 vs 320±20 min |
| Zhong et al., 2012 /  RCT [27] / RCT | DEX: 20 / 55.8±10.4 yr  SAL: 20 / 56.9±12.7 yr | Severe trauma patients | 1–2 µg/kg for 10 min then 0.2–0.7 µg/kg/h / Trauma sedation | Midazolam (0.03–0.3 mg/kg) immediately then a continuous infusion at dose of 0.03–0.2 mg/kg/h |
| Zhu, 2012 [28] / RCT | DEX: 28 / 56.2±8.2 yr  SAL: 28 / 55.7±9.2 yr | One lung ventilation /  172.5±25.6 vs 175.8±23.1 min | 2 µg/kg for 10 min then 0.3–0.5 µg/kg/h / Intraoperative | Midazolam (0.05 mg/kg), sufentanil (2 µg/kg), propofol (2–4 mg/kg), etomidate (0.1 mg/kg), vecuronium (0.1 mg/kg) / 196±28 vs 192±23 |

Abbreviations: DB, double-blind; DEX, dexmedetomidine; hour, h; ICU, intensive care unit; kg, kilogram; µg, microgram, mg; milligram; min, minutes; PROP, propofol; SAL, saline (control), SB, single blind; RCT, randomized controlled trial; yr, years

| **Table S2: Risk of bias assessment in the included studies** | Other bias | Selective reporting | Incomplete outcome data | Blinding of outcome assessment | Blinding of participants/personnel | Allocation concealment | Random sequence generator |
| --- | --- | --- | --- | --- | --- | --- | --- |
| Bekker et al., 2013 [14] | L | L | L | U | L | L | L |
| Kang et al., 2013 [15] | L | L | L | U | L | L | L |
| Kim et al., 2014 [16] | L | L | L | U | H | L | L |
| Liu and Qian, 2013 [17] | L | L | L | U | H | U | L |
| Naquib et al., 2013 [18] | L | L | L | U | L | L | L |
| Tasdogan et al., 2009 [19] | L | L | L | U | H | L | L |
| Ueki et al., 2014 [20] | L | L | L | U | L | L | L |
| Venn et al., 2001 [21] | L | L | L | U | L | L | L |
| Wang et al., 2014 [22] | L | L | L | U | L | L | L |
| Yacout et al., 2012 [23] | L | L | L | U | L | L | L |
| Zhang and Zhang, 2013 [24] | L | L | L | U | H | U | L |
| Zhang et al., 2012 [25] | L | L | L | U | H | U | L |
| Zhang et al., 2014 [26] | L | L | L | U | H | U | L |
| Zhong et al., 2012 [27] | L | L | L | U | H | U | L |
| Zhu, 2012 [28] | L | L | L | U | H | U | L |
| Legends: H: high risk; L: low risk; U: unclear risk | | | | | | | |
